# A comprehensive review on computational metabolomics: Advancing multiscale analysis through *in-silico* approaches

**DOI:** 10.1016/j.csbj.2025.07.016

**Published:** 2025-07-13

**Authors:** Mohamed S. Nafie, Abdelghafar M. Abu-Elsaoud, Mohamed K. Diab

**Affiliations:** a Department of Chemistry, College of Sciences, University of Sharjah, Sharjah 27272, United Arab Emirates; b Bioinformatics and Functional Genomics Research Group, Research Institute of Sciences and Engineering (RISE), University of Sharjah, Sharjah 27272, United Arab Emirates; c Department of Biology, College of Science, Imam Mohammad Ibn Saud Islamic University (IMSIU), Riyadh 11623, Saudi Arabia; d Pest Physiology Department, Plant Protection Research Institute, Agricultural Research Center, Giza 12311, Egypt

**Keywords:** Computational metabolomics, Drug discovery, Pharmacokinetics, Pharmacometabolomics

## Abstract

Computational metabolomics will be established in drug discovery and research on complex biological networks. This field of research enhances the detection of metabolic biomarkers and the prediction of molecular interactions by combining multiscale analysis with *in silico* and molecular docking methods. These include nuclear magnetic resonance, mass spectrometry, and innovative bioinformatics, which enable the accurate generation and characterization of metabolomes. Molecular docking is a crucial tool for simulating the interaction between ligands and receptors, thereby facilitating the identification of potential therapeutics. It also discusses the potential of metabolomics to inform drug modes of action, from pharmacokinetics to forecasting toxicity, thereby streamlining drug development pipelines. We highlight applications in anticancer, antimicrobial, and antiviral drug discovery and explain how these computational models can accelerate target validation and enhance the accuracy of therapeutic strategies. In addition, this review addresses the current challenges and future directions for computational techniques in conjunction with experimental data to advance personalized medicine. In conclusion, this review aims to highlight the prospective approaches of computational metabolomics and molecular docking that identify evolutionary adaptive metabolisms of multiscale biological systems through their synergistic utilization to overcome the key hurdles involved in both drug discovery and metabolomic research.

## Highlights

• Metabolomics integration with *in-silico* approaches for effective drug design.
• Fusion of analytical platforms for biomarker identification reliability.
• Opportunities to discover new therapeutic drugs at the molecular level.
• Predictive, cost-effective drug discovery to forecast metabolic pathway interactions.
• Understanding of metabolic variations across diseases and therapeutic strategies.

## Graphical Abstract

**Figure:**
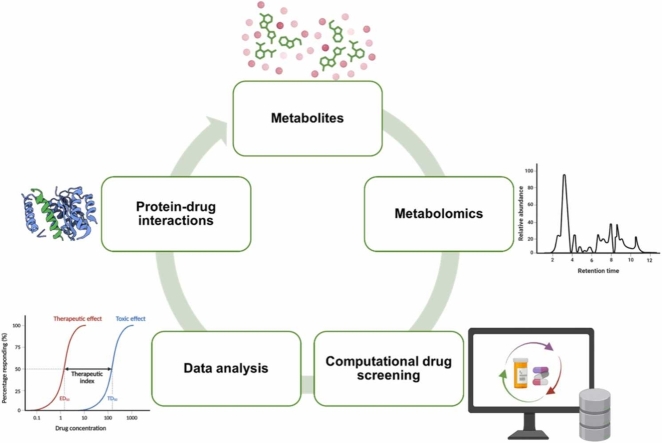

## 1 Introduction

The field of drug discovery encompasses nonclinical and clinical research studies conducted to develop new drug molecules, identify new therapeutic targets, and improve drug efficacy and safety [1]. It relies on multiple state-of-the-art methodologies, including genomics, transcriptomics, proteomics, metabolomics, phenomics, and high-throughput screening, to validate therapeutic target pathways, drug bioavailability, efficacy, and safety [2]. These validations result from interactions with those targets or other molecular targets, interactions with biological fluids, and off-target interactions, as well as common mechanisms of toxicity such as genotoxicity, reproductive and developmental toxicity, and endocrine disruption [3]. Metabolic phenotyping and metabolomics represent two robust methodologies in drug discovery that are used to define the interactions among living systems, drugs, and their target networks in terms of physiological, pathological, and toxicity effects [4].

The purpose of this field is to examine metabolisms of living organisms using a variety of analytical methods, with a special emphasis on both the short-term influence of pathophysilogical activities and the long-term influence of drugs in treatment, and to search for metabolic biomarkers reflecting the two effects [5]. Several drug-related metabolic pattern alterations, consistent with both therapeutic effects and toxicity, have been detected across multiple metabolomic analyses and metabolic phenotyping studies involving diverse classes of drugs in cellular, animal, and human model systems [6]. The development and application of such accurate, reproducible, and sensitive methodologies have enabled landmark discoveries in characterizing the mechanisms of action and pathways of adverse drug reactions during lead discovery [7]. This review introduces the primary methodologies and techniques involved in metabolic phenotyping and metabolomic studies in drug discovery, followed by their applications in assessing the pharmacological and toxicological effects of drugs in the quest to identify potential novel therapies for antidiabetic, anti-inflammatory, antimicrobial, antiviral, and anticancer applications.

### 1.1 Definition and scope

Metabolomics has emerged in recent years as a crucial tool in drug discovery, with applications in toxicology, biomarker identification, and the study of drug action mechanisms and stability [1], [8]. It involves a comprehensive study of the complete set of metabolites of a particular microorganism, cell, tissue, or organism [9]. Ultimately, metabolomics aims to capture the dynamic or steady-state changes that occur under a given condition and is comparable to the field of transcriptomics in terms of its goals, as well as similarity in the experimental methodology employed [10]. Metabolomics has been added to the toolbox of omics and is also interpreting the effects of genes and proteins in the context of current diseases or drug action [10], [11]. Although its focus varies from conventional clinical chemistry to global metabolic profiling, it intends to create a clearer understanding of the actions and functions of genes [8]. To be overly specific in its current definition might be detrimental to science [12]. In addition, methods now applied in metabolic phenotyping have led to a resurgence of drug discovery using endogenous catabolites, which hitherto met with little or no success [13]. Appropriate metabolic phenotyping and pharmacometabolomics will enable the identification of potentially toxic, ineffective, or adverse events at an earlier stage of development [14]. Furthermore, it has been calculated that an early indication of the principal causes of the metabolic syndrome could save health by a significant amount annually, thus providing substantial commercial opportunities [15].

In essence, improved understanding and potential application of the metabolism of both xenobiotic chemicals and endogenous catabolites, as well as the interplay with naturally occurring genetic and environmental factors, can be exploited *in vitro* [16]. This synergy can be exploited in drug discovery and development, for example, offering the promise of new diagnostics and prognostics [2]. Additionally, the metabolomes can provide or be mined for therapeutic targets [17]. Diverse classes of chemicals associated with particular metabolic pathways can, therefore, be identified using advances in analytical tools now applied to drug discovery, including pharmacokinetics, profiles, and toxicology [10]. This advance is significant since metabolism governs the properties of a medicinally active molecule; the route of administration, duration of action, and the possibility of active metabolites can all be manipulated [18]. Metabolomics and metabolic phenotyping also provide tools in the emerging field of systems biology, specifically the biological interpretation of large, multi-omic datasets, as the metabolome is an output of the functions of the genome and proteome [19]. Metabolomics-driven systems biology facilitates the understanding of mechanisms of action and chemical reactivity, enabling target validation processes and associated costs to be significantly reduced, thereby reducing the number of withdrawn drugs [2]. Knowledge derived from the applications of metabolomics provides a sound biological foundation for next-generation therapeutic and biomarker strategies, focusing primarily on the functioning of genes and their proteins, as well as the translation of their functions into the characteristics of specific tissues or disease states [20].

### 1.2 Historical development

The practice of medicine and the use of medicinal agents are among the oldest human endeavors [21]. In the earliest days, many drugs were obtained from the plant kingdom, although other sources, such as molds, were also used [22]. In recent years, the metabolic origins of many plant-derived agents have been elucidated, and a wide variety of metabolites of microbial origin have been characterized [23]. However, the isolation of active principles of known medicinal agents has been very inefficient. When agents were isolated and found to have the desired effects, very little information was available about the routes and the quantitative pattern of their metabolism [24]. In general, it was felt that identifying the principal excretion products of xenobiotics in animals was required as part of the process to understand the possible metabolism and, thus, the biotransformation process [25]. Particular attention has been devoted to investigating the role of hepatic biotransformation in modifying the pharmacological activity of xenobiotics [26]. In parallel with the development of biological and then clinical testing of potential xenobiotics, away from the use of models, and the advancement of more modern synthetic chemistry, our chemical understanding of the metabolism and eventual disposition of xenobiotics has progressed in direct relationship to the development of novel techniques [27]. These include purely instrumental methods such as gas chromatography, mass spectrometry, and nuclear magnetic resonance spectroscopy [28], because the development of such techniques has enabled all or parts of complicated metabolic processes to be elucidated using a wide range of isotopically labeled substrates, particularly through *in vivo* experiments, and more recently in the era of informatics, computer modeling techniques [29].

### 1.3 Knowledge gap

Computational metabolomics has made substantial strides, but several critical challenges and knowledge gaps impede its complete incorporation into drug discovery pipelines [30]. Its major shortcoming is that it does not provide standardized data preprocessing and analysis pipelines [31]. Heterogeneous methods (e.g., NMR, GC-MS, LC-MS) and experimental setups commonly yield ambiguous outcomes, which also render cross-study comparisons and meta-analyses challenging [32]. It is also challenging to interpret machine learning models for predicting biomarkers and the effectiveness of a drug [33]. Algorithms such as random forests, support vector machines, and deep learning have known high prediction accuracy, but the biological rationale underlying computed predictions is typically unclear [34]. This black-box problem hinders regulatory acceptance and clinical confidence. Additionally, most computational metabolomics studies have small sample sizes, and validation from independent or clinical cohorts is often unavailable [35]. The integration with other omics layers, including transcriptomics, proteomics, and genomics, is still in its initial phase, and only a few tools can robustly integrate multi-omics data into consistent mechanistic understandings [36].

### 1.4 Integrative metabolomics for systemic drug impact assessment

**Fig. 1:**
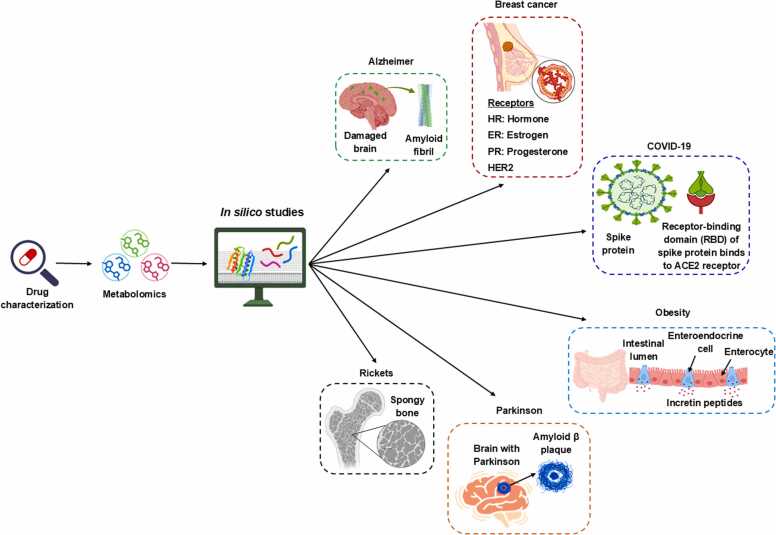
Drug characterization based on *in silico* studies in variable diseases. This figure was partially generated by Biorender (BioRender.com).

It is important to assess drug candidates against “normal” and disease tissues using metabolic phenotyping and metabolomic techniques to gain a whole spectrum view of their effects on tissues beyond their target site [37], as schemed in (Fig. 1). For example, it is not atypical for a new anticancer drug to target and selectively kill cancer cells. However, unwanted homeostatic mechanisms can occur at either this target site or at disease- or damaged sites [38]. Indeed, healthy tissues in the vicinity can suffer inadvertent damage due to the differing metabolic mechanisms between cell types, which sometimes explains the apparent side effects of drugs [39]. In cancer, this is demonstrated by comparing the cells that make up a tumor with the surrounding stroma and how the tumor recovers from treatment [40]. Such metabolism is detected by the specialized transfer of metabolites among tissues, known as “oligometastasis” [41]. Yes, not only is the drug important when testing its effect on different tissues, but also the assessment machinery itself needs to be specific to confirm localized tissue metabolic phenotyping and metabolomics assessments [42]. Metabolic phenotyping and metabolomic technologies use biofluids, cultured cells, solid tissues, and compressed tissues in a functional capacity [43]. All these (micro)environments require biofluidic and cellular reflective tools to construct the metabolic phenotype and metabolome of selected relevant tissues [44]. The complementarity of bioinformatics is a necessary component of this research, due to the inherent requirement for extracting complex data from cellular and tissue environments to understand the mechanisms of drug-induced tissue homeostasis, while avoiding the two significant issues of metabolomics in discovery biology [45].

## 2 *In-Silico* metabolomics

Metabolomics research is being transformed by *in silico* approaches, which employ computational strategies to explore complex, integrated biological systems at different scales [46]. This involves computational models, molecular docking, machine learning algorithms, and network-based approaches to examine metabolite–protein interactions and metabolic flux distributions, as well as drug–metabolite relationships [47]. Due to the vast diversity of metabolites and the complexity of metabolic pathways, conventional experimental techniques are generally unable to resolve metabolic networks [35] fully. *In silico* approaches are representative tools and cost-effective strategies that enable the detection, design, and forecasting of metabolic reactions with significantly higher accuracy [14]. Molecular docking is one of the most relevant parts of *in silico* metabolomics, which predicts interactions between metabolites and proteins, including enzymes and receptors, as well as indirect proteins involved in metabolism providing additional information relevant to enzyme kinetics, metabolic regulation, and drug discovery [48].

**Fig. 2:**
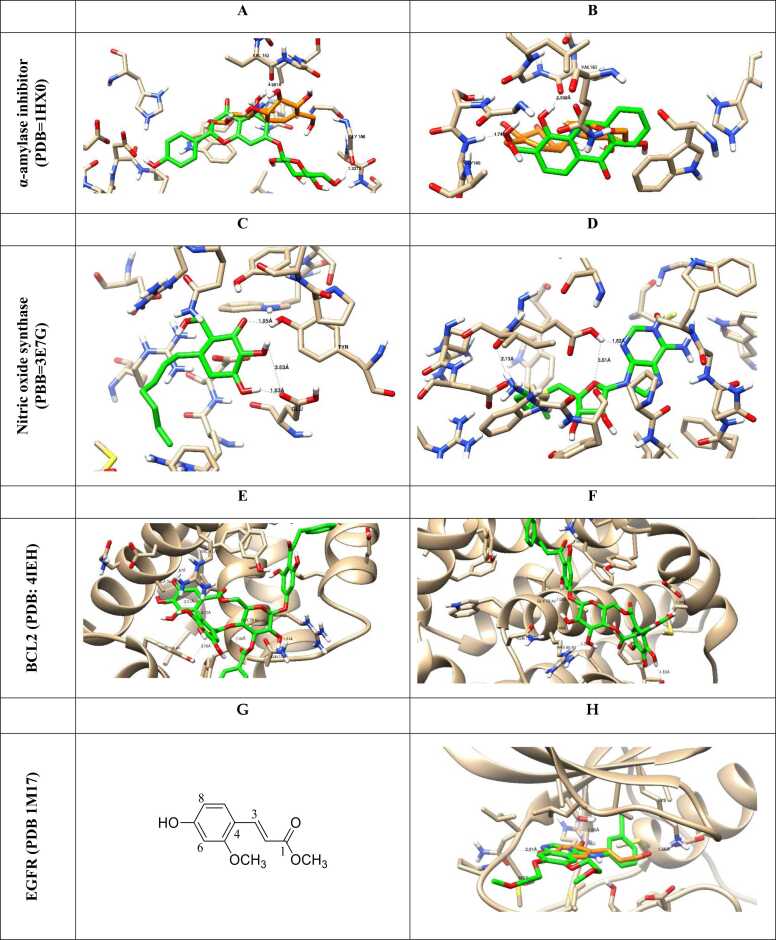

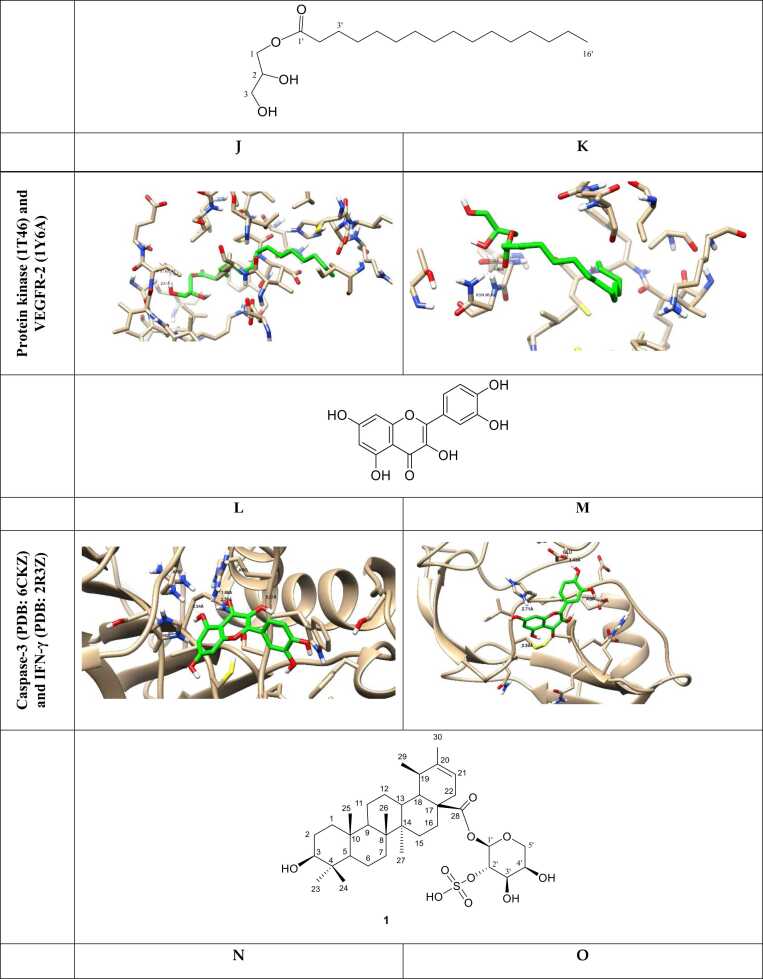

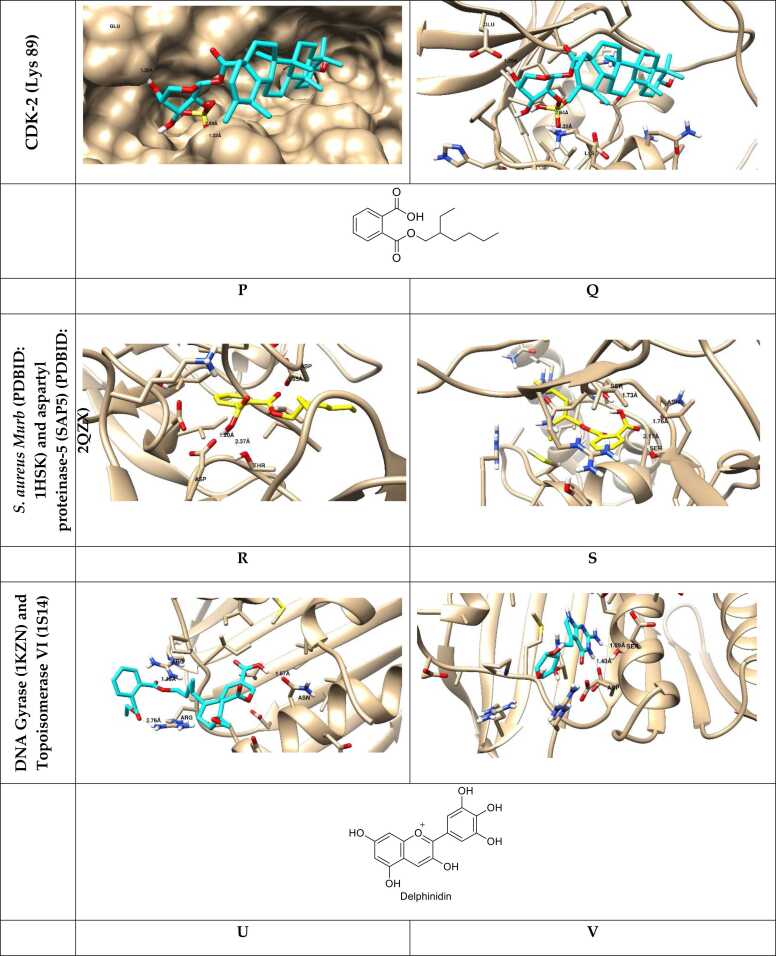

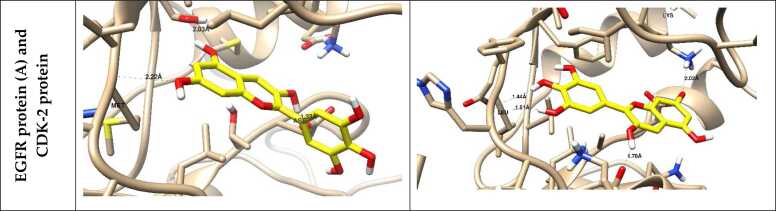
The ligand–receptor interactions of the promising docked compounds; A: Naringenin-7-*O*-glucoside, B: 1-hydroxy-2-hydroxymethyl AQ, C: Palitantin, D: S-Adenosyl-L methioninamine, E: Thonningianin-A, F: Thonningianin-B, while other compounds (G-V) are drawn inside the active site of α-amylase inhibitor (PDB=1HX0), nitric oxide synthase (PBB=3E7G), BCL2 (PDB: 4IEH), EGFR (PDB 1M17), Protein kinase (1T46) and VEGFR-2 (1Y6A), CDK-2 (Lys 89), *S. aureus* Murb (PDBID: 1HSK) and aspartyl proteinase-5 (SAP5) (PDBID: 2QZX), DNA Gyrase (1KZN) and Topoisomerase VI (1S14), EGFR protein (A) and CDK-2 protein as previously published [69], [70], [71], [72], [73], [74], [75], [76], [77].

Docking simulations predict the binding affinities of potential metabolic inhibitors and other therapeutic compounds by simulating the molecular interactions between proteins and ligands (Table 1) [49]. An integral role among these structured representations is metabolic network reconstruction, which serves to learn how metabolism is systemically regulated. Techniques like Flux Balance Analysis (FBA) and Constraint-Based Reconstruction and Analysis (COBRA) enable researchers to simulate metabolic shifts in health and disease [50]. Network-based approaches utilize metabolomic, transcriptomic, and proteomic data to identify potential metabolic vulnerabilities and biomarkers [51]. The fourth progress is powered by machine learning, which has further enriched *in silico* metabolomics-empowered predictive modeling, pattern recognition, and metabolite autoidentification [47]. In supervised and unsupervised learning methods, metabolic fingerprinting and deep-learning models are promising for studying high-dimensional metabolomic datasets [52]. In addition, by employing virtual screening approaches, thousands of compounds can be screened against metabolic targets, thereby facilitating drug discovery and repurposing [53]. With the rapid advancement of quantum mechanics (QM) and molecular dynamics (MD) simulations, these approaches have become powerful computational techniques in food metabolomics, providing in-depth mechanistic insights into enzymatic processes, metabolome stability, and molecular dynamics in biochemical systems [54]. Such simulations may aid in unraveling the dynamics between enzymes and their substrates, thereby putting us on a path to rational design of metabolic modulators [55]. *In silico* metabolomics has a variety of applications, as it can be used for precision medicine and drug development, metabolic engineering, and environmental metabolomics [10], [56], as shown in (Fig. 2). It will help identify biomarkers for early detection of disease, optimizing microbial strains for industrial biotechnology, and assessing the impact of diet and environmental toxins on metabolic health [57].

**Table 1:** *In silico* metabolomic highlighting the binding affinity of some tested natural product-based compounds against molecular targets.

**Target disease**	**Predicted therapeutic**	**PDB code studied**	**Binding energy (kcal/mol)**	**Key findings**	**Ref.**
Alzheimer	Isorhamnetin-O-rutinoside	4MOE (AChE), 4AQD (BChE)	−22.00 (AChE), −29.90 (BChE)	Exhibited the highest binding affinity with both AChE and BChE.	[59]
	Sissotrin	4MOE, 4AQD	−20.76 (AChE), −19.82 (BChE)	Demonstrated strong inhibitory potential against both AChE and BChE.	
	Diosmetin	4MOE	−19.54	Showed significant binding to AChE.	
	Rosmarinic acid	4MOE, 4AQD	−19.32 (AChE), −22.77 (BChE)	Exhibited strong interaction with both AChE and BChE.	
	Kaempferol hexoside	4MOE, 4AQD	−19.00 (AChE), −20.76 (BChE)	Showed notable affinity towards both enzymes.	
	Kaempferol−7-neohesperosides	4MOE, 4AQD	−16.99 (AChE), −23.58 (BChE)	Displayed significant binding interactions.	
	Acacetin	4MOE	−16.45	Presented promising binding properties with AChE.	
	Taxifolin	4AQD	−21.44	Showed notable binding interaction with BChE.	
	Apigenin-O-hexoside	4AQD	−19.35	Showed good inhibitory potential against BChE.	
Breast cancer	Baptifoline	4DRH	−8.80	Showed strong binding affinity against mTOR and PR receptors.	[60]
	Brevianamide F	2J6M	−8.20	Demonstrated effective binding with EGFR and mTOR receptors.	
	Odorinol	3PP0	−7.50	Exhibited strong binding with HER2 receptor.	
	Perlolyrine	4OAR	−7.30	Showed good docking affinity with PR receptor.	
	Thyronine	3ERT	−6.60	Exhibited potential interaction with ERα receptor.	
	Tyrphostin B48	2J6M	−7.80	Demonstrated notable binding with EGFR receptor.	
	Tryprostatin B	4OAR	−7.20	Indicated significant interaction with PR receptor.	
COVID−19	Sesaminol glucoside	6LZG	−10.00	Exhibited the strongest binding affinity, forming hydrophobic and electrostatic interactions with the spike protein.	[61]
	25-Hydroxyvitamin D3–26,23-lactone	6LZG	−9.90	Formed strong hydrogen bonds and hydrophobic interactions with key residues of the spike protein.	
	6′′-O-Acetylglycitin	6LZG	−9.70	Showed significant binding potential with stable hydrophobic and hydrogen bond interactions.	
	Saquinavir	6LU7	−9.50	Showed strong binding to the main protease (Mpro), suggesting significant antiviral potential.	[62]
	Ritonavir	6LU7	−8.90	Exhibited significant inhibition of Mpro activity in multiple simulations.	
	Lopinavir	6LU7	−8.70	Demonstrated good binding affinity with Mpro, highlighting its potential as a therapeutic candidate.	
	Tegobuvir	6VSB	−9.30	Showed high binding affinity with the spike glycoprotein, suggesting its role in inhibiting viral entry.	
	Eucalyptus-derived compounds	6LU7	−8.50	Demonstrated strong inhibitory potential against Mpro in *in-silico* studies.	
	Tinosponone from *Tinospora cordifolia*	6LU7	−8.20	Exhibited promising antiviral interactions.	
	Hesperidin, Rutin, Herbacetin	6LU7 and 6VSB	−8.60 to −9.10	Exhibited multitarget potential against viral proteins.	
	Glycyrrhizic acid	6VSB	−9.00	Showed significant binding with the spike glycoprotein, inhibiting virus-host interaction.	
HIV/AIDS	Cyanidin−3-glucoside	3U71	−57.89	Showed the strongest binding affinity and highest stability.	[63]
	Maslinic acid	3U71	−48.13	Exhibited strong inhibition potential against HIV−1 protease.	
	Corosolic acid	3U71	−43.90	Demonstrated favorable binding and stability with HIV−1 protease.	
	Betulinic acid	3U71	−43.74	Showed good affinity and interaction stability.	
	Oleanolic acid	3U71	−42.01	Exhibited strong binding and compactness within the active site.	
	Ursolic acid acetate	3U71	−40.65	Displayed significant binding potential with stable interactions.	
Lung cancer	Catechin	2A4L (CDK−2), 1M17 (EGFR)	−16.39 (CDK−2), −18.69 (EGFR)	Demonstrated strong inhibition potential towards CDK−2 and EGFR proteins, enhancing apoptotic pathways.	[64]
	Methyl gallate	2A4L (CDK−2), 1M17 (EGFR)	−15.79 (CDK−2), −18.25 (EGFR)	Showed high binding affinity with CDK−2 and EGFR, supporting apoptosis induction.	
	Delphinidin	2A4L (CDK−2), 1M17 (EGFR)	−16.22 (CDK−2), −17.55 (EGFR)	Exhibited promising interaction with both proteins, leading to apoptotic effects.	
	Isorhamnetin	2A4L (CDK−2), 1M17 (EGFR)	−14.89 (CDK−2), −16.98 (EGFR)	Showed significant binding energies, contributing to cancer cell cycle arrest.	
	Euscaphic acid	2A4L (CDK−2), 1M17 (EGFR)	−15.26 (CDK−2), −17.44 (EGFR)	Effectively targeted both proteins, aligning with its observed anticancer activities.	
	β-Sitosterol Glucoside	2A4L (CDK−2), 1M17 (EGFR)	−14.78 (CDK−2), −16.85 (EGFR)	Exhibited high affinity, suggesting potential as an anticancer agent.	
Obesity	Epigallocatechin−3-gallate	3TOP	−8.19	Exhibited strong binding affinity with α-glucosidase, suggesting anti-obesity potential.	[65]
	Myricetin−3-O-rhamnoside	3TOP	−8.47	Demonstrated comparable binding affinity and stability.	
	Desmanthin−1	3TOP	−6.41	Showed moderate binding interactions with α-glucosidase.	
	Luetic acid	3TOP	−5.06	Presented a reasonable binding potential, indicating potential activity in metabolism regulation.	
	Gallic acid	3TOP	−3.47	Exhibited lower binding affinity yet demonstrated inhibitory potential.	
	Protocatechuic acid	3TOP	−3.82	Showed low binding affinity but may contribute to synergy with other compounds.	
Parkinson	Epicatechin-gallate	6G54	−8.50	Exhibited the highest binding affinity with ERK2, stabilizing key interactions.	[66]
	Hesperidin	6G54	−8.40	Demonstrated strong binding with ERK2, forming stable hydrogen bonds.	
	Baicalein	6G54	−6.40	Formed strong hydrophobic and hydrogen bond interactions with ERK2 residues.	
	Naringin	5NU5	−8.40	Exhibited high binding affinity with EP300, indicating potential therapeutic effects.	
	Taxifolin	5NU5	−7.90	Formed strong hydrophobic interactions with EP300, suggesting neuroprotective roles.	
	Mangiferin	5NU5	−7.80	Showed notable stabilization in EP300 binding, indicating strong interaction potential.	
	Hesperidin	5NLK	−7.90	Showed significant affinity towards CREBBP, stabilizing through multiple bonds.	
	Rutin	5NLK	−6.60	Exhibited good binding energy with CREBBP, supporting its potential as a therapeutic candidate.	
Rickets	D-Bishomo−1α,25-dihydroxyvitamin D3 analog	8PZB	Not specified	Exhibited similar binding affinity and interaction patterns to the native hormone calcitriol.	[67]
	19-nor vitamin D3	8PZ8	Not specified	Formed comparable hydrogen bonds to calcitriol but with some differences in side-chain orientation.	
	2-Methylene−19-nor vitamin D3 analog	8PZ9	Not specified	Showed enhanced stabilization due to additional side-chain interactions.	
	2-Hydroxypropylidene−19-nor vitamin D3 analog	8PZ6	Not specified	Formed stronger hydrogen bonds, particularly with Arg302, suggesting improved receptor affinity.	
	2β-Methylated vitamin D3 analog	8PZ7	Not specified	Showed weaker interactions, correlating with its lower biological potency.	
	ω-aminoalkoxylxanthone	4URN	−6.80 to −7.30	Exhibited strong binding affinity with bacterial topoisomerase IV, inhibiting bacterial DNA replication, indicating potential antibacterial activity.	[68]
	Ciprofloxacin	4URN	−7.80	Effectively inhibited bacterial DNA replication *via* strong interaction with the enzyme.	
	Novobiocin	4URN	−7.20	Showed moderate binding affinity, confirming its known activity.

Combining computational approaches with experimental metabolomics has accelerated discovery in systems, synthetic biology, and pharmacology. It can be used to design targeted therapeutic approaches rationally, as visualized in (Fig. 2). The continuously improved computational power will lead to the next-generation *in-silico* metabolomics toward a combination with AI, big data analytics, and high-throughput experimental technology, which offers a breakthrough in metabolic model accuracy and predictive power [47]. Computational metabolomics, coupled with experimental methods, will be critical for driving discoveries in biomedical research and gaining deeper insights into metabolism and its role in health and disease [58]. As summarized below, in (Table 1), there is a list of identified natural compounds through the metabolomics approaches, in which these compounds exhibited promising binding affinities towards their respective molecular targets in biological activities.

Molecular representation of the docked compounds inside their respective targets using the molecular docking studies in ligand-receptor interactions and the surface views using the 2D and 3D models, in which the docking compounds exhibited good binding interactions with the key amino acids for the target activity [59], [60], [61], [62], [63], [64], [65], [66], [67], [68].

## 3 Multiscale computational approaches in metabolomics in drug discovery

**Fig. 3:**
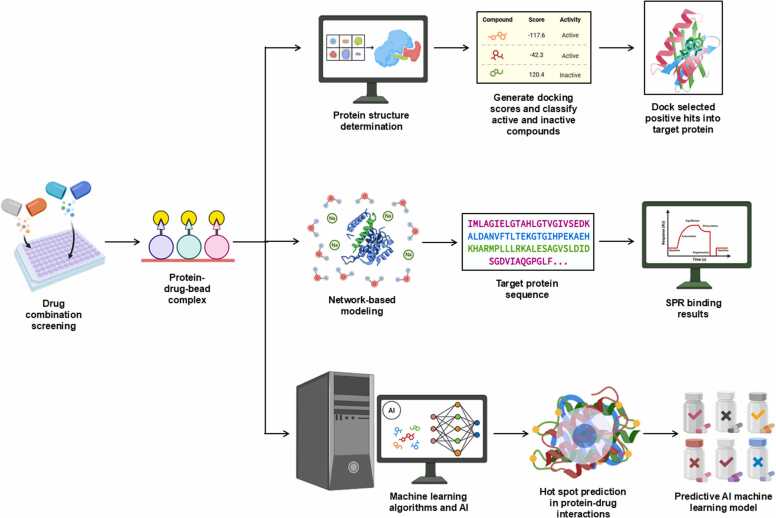
Computational techniques based on metabolomics for drug discovery. This figure was partially generated by Biorender (BioRender.com).

Computational metabolomics is the application of high-throughput metabolomic data, combined with advanced computational methods, to identify biomarkers, drug targets, and outcomes [78]. Historically, metabolomics has been limited to compound identification and pathway assignment through database searches, such as KEGG, MetaCyc, and HMDB [79]. Nevertheless, the field has expanded to comprise a set of *in silico* practices such as molecular docking, genome-scale metabolic model flux balance analysis, machine learning, and multiscale simulation, which allow scientists to study complex metabolic networks and to simulate drug-metabolite interactions *in silico* [80], as mentioned in (Fig. 3). An early landmark study in this area was conducted by Wishart and colleagues, who created the Human Metabolome Database (HMDBs), a key resource for chemical annotation and pathway mapping [81]. At the same time, Nicholson and coworkers demonstrated the potential of NMR-based metabolic profiling in the investigation of xenobiotic metabolism and its importance for drug-induced toxicology [82]. These initial attempts set the stage for the potential implementation of metabolomic phenotypic profiles in the diagnosis and prediction of drug responses.

### 3.1 Seminal applications in drug development

An example of the application of computational metabolomics is the work of Dasgupta *et al*. (2023), who based their prediction on techniques of metabolic profiling to differentiate between two common progressive lung diseases, asthma and chronic obstructive pulmonary disease (COPD) [83]. Through the integration of nuclear magnetic resonance (NMR), gas chromatography-mass spectrometry (GC-MS), liquid chromatography-mass spectrometry (LC-MS), and multivariate statistical analysis, the study detected lipid mediators and amino acid derivatives associated with therapeutic efficacy. Therefore, stratified therapy can now be tailored according to metabolic phenotypes [83]. In the field of antiviral drug development, Soma and Perera (2024) investigated the effect of oseltamivir in influenza patients using LC-MS-based untargeted metabolomics [84]. Their results revealed marked changes in purine and pyrimidine metabolism pathways, as well as changes in urine metabolites in these pathways, as determined by principal component analysis (PCA) and pathway enrichment. The data followed a decrease in viral loads [84]. This work provided examples of how metabolomics could be used to reveal direct antiviral action as well as the host metabolic response [83], [84].

### 3.2 Docking tools and techniques

Data analysis of metabolomics data typically involves several tools with specific analytical purposes. For example, molecular docking tools such as AutoDock and SwissDock are widely used for estimating the binding affinities of putative metabolites with protein targets [85]. Reconstruction and simulation of metabolic networks are currently performed by tools such as the COBRA Toolbox for MATLAB and MetaFlux, which enable investigation of metabolic fluxes as a system in response to drug perturbation [86]. During the same time, machine learning techniques, as provided in frameworks such as DeepChem and Scikit-learn, are also used to conduct biomarker identification, toxicity risk assessment, and drug activity profiling using complex metabolic signatures [87]. These tools have significantly enhanced the resolution and predictive capacity of metabolomics studies in pharmaceutical science (Fig. 3), yet each comes with inherent strengths and limitations, which are summarized in Table 2.

**Table 2:** Summary of computational techniques in metabolomics for drug discovery.

**Technique**	**Application**	**Key Tools**	**Strengths**	**Limitations**	**Ref.**
Molecular docking	Ligand-target prediction	AutoDock, GOLD, SwissDock	Structural insight, fast screening	Accuracy depends on protein structure data	[88]
Network-based modeling	Pathway simulation, target ID	COBRA Toolbox, MetaFlux	Mechanistic interpretation	Requires curated metabolic models	[86]
Machine learning and AI	Biomarker discovery, drug response	DeepChem, scikit-learn	Handles high-dimensional data	Needs large, labeled datasets	[89]
Multiscale modeling	System-wide simulation	CellDesigner, COPASI	Cross-scale integration of omics data	Computationally intensive	[90]

## 4 Metabolomics in anticancer drug discovery

From the various basic research fields in anticancer drug discovery, the role of metabolomics, applied either alone or in combination with metabolic phenotyping, has experienced significant growth in the last two decades [29]. Some of these anticancer metabolomic studies offer insight into the alteration of amino acid metabolism during tumorigenesis [91]; others provide clues about lipidome alteration due to the tumor environment and are associated with enhanced cell proliferation or unveil the role of sialylated *N*-glycans as a bioprocess for detection of cancer cells in response to altered glycomic patterns overexpressing sialyltransferase during tumorigenesis [92]. However, the high-throughput and high-sensitivity applications of these metabolites, through the generation of fingerprints associated with tumor diagnosis and progression, play a key role from a molecular point of view in translating these data into cancer diagnosis and personalized therapy treatments in the clinical arena [93], [94].

### 4.1 Overview of anticancer drug development

Cancer is a complex genetic disease defined as the uncontrolled, abnormal division and growth of cells in the human body [95]. A wide array of genetic changes in tumorigenic tissues plays a crucial role in tumorigenesis, making the recovery of cancer cell phenotypes quite complicated [96]. This, along with resistance to most anticancer agents, is a major obstacle in developing effective cancer treatments and the successful discovery of new anticancer drugs [97]. Global metabolic changes render these extreme phenotypes ineffective. Metabolic phenotyping finds its application in drug discovery through the analysis of small molecules, focusing on metabolic pathways to highlight the differences between healthy and pathological cellular processes that could be targeted with novel drugs [98]. Metabolic phenotyping approaches include metabolomics, lipidomics, and fluxomics, with the latter two being the most commonly employed today in understanding the metabolic basis underlying new compound activity [99].

Nowadays, anticancer drugs target enzymes in metabolic pathways crucial for tumorigenesis [100]. More recent developments have led to inhibitors of receptor tyrosine kinases, MAPK-ERK pathway inhibitors, and mammalian target of rapamycin pathway inhibitors, among many others [101]. The clinical success and availability of a wide array of anticancer drugs, coupled with the increasing costs of drug discovery, have prompted researchers to focus on novel methods and strategies aimed at a deeper understanding of cancer metabolism [29]. Metabolic phenotyping has applications in drug discovery through the analysis of small molecules, focusing on the metabolic pathways essential for cancer cell survival [98]. The genetic heterogeneity, together with the capacity to adapt to changes in blood levels of substrates, ions, and growth factors, makes it challenging to suppress cancer cell growth and resistance to death [102]. Animal and human tumors are spatially heterogeneous, adding layer of complexity to the discovery of personalized cancer treatments [103].

### 4.2 Metabolomic biomarkers in cancer research

In both diagnosis and treatment stratification, cancer of unknown primary origin (CUP) presents both a diagnostic challenge and a marker-based dilemma [104]. Studies have paved the way for potential clinical applications based on the association of these markers with the underlying tumor biology [105]. They were reported to be cell-enter proteins, tumor cell-secreted proteins with unknown functions, and other serum protein peaks of colon cancer cells, respectively, which indicated that this method can detect approximately 25 peptides of lung cancer with high sensitivity, and 24 of them are accurately diagnosed [106].

Emergency detention orders (EDOs) add the potential to identify and classify underlying cancer types when positive samples are found [107]. These proteins provide a selective advantage for cells that are present and have, therefore, become established biomarkers for cancer diagnosis [108]. However, proteomic approaches have been successful in some tissues, and mass spectrometry studies in blood have identified several serum protein markers, including Fab fragment heavy chain-binding antigen, migration inhibitory factor, and transitional cell carcinoma-specific antigen [109]. Based on the serum protein signatures and the identified secretome-derived proteins detected from the two groups of cells, a new specific set of metabolomic phenotypic markers discriminating sesquiterpenes of the two cells together with a unique combination of the markers was established. Metabolomic profiling detected 28 essential metabolites [110]. They include 15 glycerophospholipids, six amino acids, five carnitines, and two acylcarnitines, which performed at their ideal accuracy level for discriminating between healthy patients and patients with non-small cell lung cancer in cell culture medium [110].

### 4.3 Metabolomics approaches for drug screening

Metabolomics is an innovative yet complementary approach to traditional genomics and proteomics [29]. Considering the complexity of human metabolic networks, this approach provides a comprehensive snapshot of cellular metabolic status [17]. With recent developments in analytical methods, such as high-performance chromatography, gas chromatography, and mass spectrometry, metabolic changes due to various conditions can be effectively detected and quantified [111]. Since alterations in human metabolism are highly correlated with the onset and progression of various diseases, studying the small molecules that comprise the metabolome is highly attractive. As a result, metabolomics and metabolic phenotyping can be especially valuable in discovering new treatments and drug discovery [10]. Simultaneously, the insights obtained into the potential mechanisms can bring significant novelty to drug design and discovery processes [2]. This review will explore and discuss the role of specialized metabolic profiling in drug development targeting anticancer, antidiabetic, and antimicrobial activities, as well as anti-obesity research.

The technology platforms used in metabolomics can screen for a broad range of low-molecular-weight compounds, including amines, amino acids, fatty acids, steroids, carbohydrates, and organic acids [112]. These reasonably sensitive techniques provide a comprehensive view of chemistry obtained from a biological matrix, often combining mass spectrometry with liquid or gas chromatography to separate compounds prior to analysis [113]. Fingerprint and targeted analysis can be done using several commercially available data processing platforms [114]. Profiles from different matrices obtained *via* metabolomics can be used to evaluate the safety and efficacy of pharmaceutical and nutraceutical products [115]. Initial screening of drugs in development is conducted in cell cultures and then in animals, followed by human clinical trials. Metabolomics may have several applications in drug discovery [1].

## 5 Metabolomics in antimicrobial drug discovery

Metabolomics and metabolic phenotyping remain promising scientific disciplines within the drug discovery and development arena [14]. These methods contribute to a better understanding of the mechanism of drug action and potential drug toxicity, reveal novel targets for therapeutic intervention, facilitate hit selection and lead optimization, and contribute to patient stratification and the development of personalized treatment regimens [116]. Antibiotic resistance represents a serious public health challenge [117]. To address this threat, new antimicrobials and new classes of antibiotics must be discovered at a faster pace. In this chapter, we specifically cover studies employing metabolomics methods within the area of antimicrobial drug discovery. Metabolomics approaches are employed to address topics including the discovery of new antimicrobial agents, the deciphering of the mode of action of antimicrobial agents, the determination of mechanisms of antimicrobial resistance, the identification of potential new targets for antimicrobial treatment, the classification and identification of microorganisms, and determining the structure of microbial metabolomes [118], [119]. Metabolomic approaches can be applied using different microbial cell models, including bacteria or fungi [120].

### 5.1 Challenges in antimicrobial drug development

Bacterial infections are the major cause of hospital admissions and are responsible for high rates of morbidity and mortality [121]. It is a public health issue of increasing concern due to the emergence of antibiotic resistance [117]. There is an urgent need for innovative research to combat this global challenge [122]. In the last few years, the development of new antimicrobial drugs has demonstrated a significant reduction [123]. Bacterial cell metabolism differs significantly from host metabolism; therefore, inhibiting host-pathogen competition is a target for further antimicrobial drug development [124]. To discover the bioactive microbial metabolites responsible for these activities, antibiotic screening and the identification of their mechanisms are practical approaches [125]. This information can be obtained using a metabolomics approach combined with metabolic phenotyping analysis [126]. Moreover, the search for new antibiotics has been negatively impacted by the rediscovery of known compounds, mainly the classical antibiotics, tetracyclines, and phenotyping groups of this class of antibiotics, which was historically completed under a limited survey of actinomycete genera [127]. Currently, only a tiny fraction of the natural product biosynthetic gene clusters in the environment are recruited to be activated for the synthesis of metabolites, resulting in the majority of unexplored biosynthetic gene clusters becoming cryptic clusters that could represent a rich reservoir for the discovery of new bioactive metabolites [128]. Additionally, this feature increased the contribution toward the presence of potent enzymatic activity and other bioactive compounds in the antibiotics’ mode of action [129].

### 5.2 Metabolomics for antibiotic discovery

The development of effective antimicrobial treatments has played and continues to play a crucial role in maintaining an adequate public health system [130]. The current situation of increasing numbers of multi-resistant pathogens makes the discovery and development of new antibiotics in this field critical to overcoming the lack of efficient treatments caused by these pathogens [131]. Metabolomics techniques, including specific metabolic phenotyping approaches, can provide rapid information and insights that save time and resources in the discovery of new antibiotics [132]. These approaches can provide valuable and rapid information on the metabolic effects of antibiotics at the cellular level, offering a valuable resource for proposing and/or optimizing new therapeutic compounds that act against a wide variety of human pathogens [116].

The term antibiotic refers to any substance that can kill bacteria or, in a more general sense, inhibit their growth through metabolic effects [133]. However, the discovery and development of new antibiotics have declined since the golden years of the pharmaceutical industry, from the 1940s to the 1990s [134]. The emergence of multi-resistant bacteria has revealed the insufficiency that can be generated by an over-reliance on the routine use of a few antibiotic families [135]. Bacteria possess a high mutational capacity, enabling them to evolve resistance mechanisms against these compounds rapidly. Furthermore, the overuse of these treatments accelerates the processes of resistance development [136]. The interactions between bacterial cells and between these cells and their environment are metabolic. They result from trafficking signaling when these molecules are derived from bacterial metabolism. This is an excellent hint for developing new therapeutic strategies [137].

### 5.3 Metabolic pathways as drug targets

The discovery of new mechanisms or leads in treating diseases should reduce the growing burden on society [130]. Metabolic pathway knowledge can offer insights into new and improved drugs [138]. In recent years, human metabolome projects have found a potentially large number of metabolites likely associated with specific phenotypes [17]. These metabolites have elucidated important pharmacokinetic and pharmacodynamic properties in humans, which are crucial for the successful progression of drug discovery programs to larger clinical trials [139].

Inhibition of enzymes within a pathway can lead to a buildup of the metabolite and the depletion of the metabolite at the distal end of the pathway [139]. The accumulation of para-aminobenzoic acid (PABA) and depletion of folate result in a limited cellular capacity to synthesize purine and thymidine [140]. Bacterial turnover of PABA to folate is catalyzed by two reactions: initial activation by dihydropteroate synthase and subsequent amino acid conjugation by dihydrofolate synthase [141]. After folate biosynthesis, the genome sequence contains numerous other enzymes involved in biosynthesis, and the pathway is associated with a salvage function to mitigate drug interference [142]. Natural metabolic functions could aid the rationalization of drugs that interfere with these pathways [143]. Drug resistance mutations can be predicted from the high-resolution structure of substrate-binding domains [144].

## 6 Metabolomics in antiviral drug discovery

**Fig. 4:**
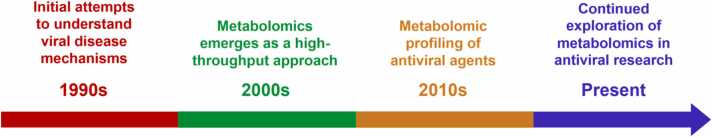
Advancements in antiviral drug development through metabolomics.

Integrating metabolomic data with preclinical virology research opens a vast opportunity for a systems-level understanding of host-pathogen interactions [145]. Moreover, the metabolomic approach is essential for studying the pathophysiological changes linked to viral infection [116]; identifying unique metabolites or pathways pivotal in disease progression is important for the development of novel diagnostics and for understanding the mechanisms associated with viral infections [122], as mentioned in (Fig. 4). In drug discovery, the application of metabolomics has been beneficial for evaluating therapeutic efficacy and for investigating the pharmacological mechanisms of antiviral agents [146]. The LC-MS-based metabolic profiling may identify the downregulation of viral replication-related metabolites upon treatment with antiviral drugs, including oseltamivir or remdesivir [147]. This shift can be calculated by a multivariate statistical analysis procedure such as PCA or orthogonal partial least squares-discriminant analysis (OPLS-DA) that discriminates the metabolic phenotype of treated versus untreated samples [148]. Pharmacological mechanisms have further been inferred by overlaying affected metabolites on host metabolic pathways and identifying enzymatic targets or pathway perturbations. These interpretations serve to confirm drug effects and to characterize potential off-target effects or resistance mechanisms [149]. In this review, we address the metabolomic applications for developing new antiviral agents, emphasizing the role of nucleobase/nucleoside analogs in antiviral drug discovery.

In the context of metabolomic data production, the potential biological findings from antiviral (or other drug) experiments are broad, covering drug modes of action, safety effects, drug resistance development, and biomarker findings [150]. First, metabolomics is considered a high-throughput approach, meaning 10–1000 s of metabolite concentrations can be determined in the same experiment [145]. Second, metabolomics has the advantage of representing the end-point phenotype of a biological system [151]. Plasma, serum, urine, cerebrospinal fluid, and tissues such as the liver and kidney are frequently used as experimental materials for metabolomic analysis in drug development research [152].

### 6.1 Current challenges in antiviral drug development

Targeting the viral life cycle and host-virus interactions using small molecules or other modalities can be an effective strategy for discovering antiviral agents [153]. Despite developing potent drugs with successful patient outcomes, there is a never-ending race against viruses due to the rapid emergence of drug-resistant variants, antigenic shifts leading to vaccine escape, and the frequent occurrence of pandemics [154]. Thus, innovative drug development is essential in this field to overcome the aforementioned challenges [155]. This chapter introduces some challenges in antiviral drug development and their impacts on human health during an infectious outbreak.

These antivirals have emerged from various studies that rely on the knowledge of the structure and function of specific viral and host proteins, as well as the molecular interactions that occur within infected cells [156]. This knowledge, along with the subsequent development of antiviral therapeutic agents and treatment strategies, can help manage viral pandemics effectively [157]. Nonetheless, several antiviral drugs are currently available that have been in use for many years in clinical settings [158]. These established therapeutics, together with modern inhibitors, are also promising tools that can help unravel some of the molecular mechanisms of virus replication and cellular behavior [159].

### 6.2 Role of metabolomics in viral infection studies

Viral infections are a significant health issue worldwide and are associated with substantial healthcare expenses [160]. Viral infections can be acute or chronic, resulting in serious long-term health issues [161]. Metabolomic studies of viral infections, diabetic complications following infection, and therapeutic targets for viral diseases are crucial for the human population [146]. Metabolomic research aims to explore host responses to infection or viral diseases and various complications synoptically [162]. Over the last three decades, various attempts have been made to understand the mechanisms of viral diseases using state-of-the-art technologies [191]. However, the exploration of the host response through metabolomic studies in virally infected tissues or biological fluids remains limited compared to transcriptomics and genomics [116].

Metabolomic studies for viral diseases such as influenza, HCV, human immunodeficiency virus, and dengue have been published using untargeted LC-MS and GC-MS in cell lines and serum specimens [163]. While the nature of the metabolites across different viral diseases varies, some common pathogenic mechanisms, such as suppression of the immune response, increased oxidative stress, decreased levels of tricarboxylic acid cycle intermediates, altered lipid metabolism, and a decrease in the levels of a set of essential amino acids were identified [164]. The major potential therapeutic targets for these diseases were amino acids, fatty acids, nucleotide biosynthesis, and related lipid metabolism [102]. An in-depth understanding of the host response benefits the development of drugs targeting metabolically regulated molecules as potential therapeutic options [1], [165].

### 6.3 Metabolomic profiling of antiviral agents

Metabolic profiling has been exploited to investigate and identify potential metabolic targets of antiviral agents, as well as to identify possible metabolites that can be used as efficacious pharmacodynamic markers and potential toxicity induced by antiviral drugs [138]. The global metabolomics approach has been used to explore the antiviral effect of chemotherapy against various viruses [116]. Glycyrrhizin was identified as the metabolite in the GL pretreatment group after infection, while vitamin E was increased at specific time points in the MT pretreatment group [166]. A condensate mixture of various isomers of the major chemical components of glycyrrhizin has been found to be active against a specific virus [167]. The autophagy modulator dose was associated with the addition of a compound in the presence of another virus [168].

The antiviral characteristics of extracts of wild species growing in a specific region were identified using analysis of their metabolites [169]. A model was established by extracting the metabolites from the liver and homogenizing the tissue during gastric stress, combined with analysis, and successfully applied to lifelong pharmacokinetics of antiviral prodrugs [170]. Most of the absorbed prodrugs are converted to a specific compound, and the prodrug is absorbed into the blood and metabolized by previously identified metabolic pathways, resulting in a specific compound that increases at a ratio of approximately 7:1 in most studies [171]. This study demonstrated metabolomics in a clinically relevant model characterized by fluorescence as a systemic measure of infection-induced tissue injury and imaging of the liver [172]. The research can identify potential treatment measures to reduce the high risk of a specific flu [173].

### 6.4 Metabolomics insights into nucleoside analog-based antiviral therapies

Nucleobase and nucleoside analogs significantly contribute to the blockbusters of antiviral pharmacotherapy, as they can mimic endogenous nucleotides and thus act as inhibitors of viral replication enzymes, such as RNA-dependent RNA polymerase (RdRp) or reverse transcriptase [174]. Remdesivir, ribavirin, zidovudine, and acyclovir are nucleoside analogues that are taken up by the viral nucleic acids, as a result of which chain termination or enzyme inhibition occurs [175]. The analogs can also inhibit host nucleotide metabolism due to their structural homology with indigenous metabolites, resulting in toxicity or the development of resistance [176]. Metabolomics provides a systems-level approach to study the intracellular fate, activation, and off-target effects of nucleoside analogs [177]. By targeted LC-MS/MS and isotope-tracing procedures, the conversion of prodrugs into their active triphosphate metabolites has been traced in host cells [178]. For instance, metabolomic profiling has revealed the metabolic fate of remdesivir by describing the stepwise reduction of guanosine triphosphate of remdesivir and the ability of its triphosphate form to compete with ATP for viral RNA incorporation [179].

Furthermore, metabolomics has significantly contributed to the detection of metabolic footprints associated with the toxic effects of nucleoside analogs [180]. For example, zidovudine (AZT) toxicity has been associated with mitochondrial toxicity, measurable by changes in TCA cycle intermediates and lipid metabolism [181]. High-resolution metabolomic profiling has shown that these agents lead to the accumulation of deoxynucleotide pools and perturbation of purine salvage pathways over longer periods of exposure [182]. These metabolomics-based insights in antiviral drug development are also being incorporated into computer models for pharmacokinetic simulations, the prediction of toxic effects, and the rational design of structurally related analogs with greater specificity [33], as summarized in Table 3. In this regard, metabolomics was instrumental not only in gaining an understanding of the mechanism of action of nucleoside analogs (and predicting and/or assessing their efficacy) but also in developing a predictive paradigm for the optimal use of nucleoside analogs to minimize potential toxicities and resistance [183].

**Table 3:** Summary of key nucleoside analogs, their mechanisms, and metabolomics-based insights.

**Drug**	**Chemical structure**	**Viral target**	**Mechanism of action**	**Metabolic behavior**	**Metabolomics insights**	**Ref.**
Acyclovir		Viral DNA polymerase	Chain termination in viral DNA synthesis	Activated by viral thymidine kinase to triphosphate form	Flux profiling showed selective accumulation in infected cells, limiting host toxicity	[184]
Remdesivir		RNA-dependent RNA polymerase (RdRp)	Chain termination *via* ATP mimicry	Prodrug to GS−441524 and triphosphate *via* kinases	Monitored intracellular triphosphate formation; competition with ATP tracked *via* LC-MS	[185]
Ribavirin		Viral RNA polymerase	Mutagenesis and GTP pool depletion	Converted to mono-/tri-phosphate forms; depletes purine pools	Identified disruptions in purine metabolism and increased inosine monophosphate (IMP)	[186]
Sofosbuvir		HCV RNA polymerase (NS5B)	Incorporation and premature chain termination	Prodrug activation in hepatocytes	Metabolomic tracing confirmed liver-specific activation and minimal systemic exposure	[186]
Zidovudine (AZT)		Reverse transcriptase	DNA chain termination after incorporation	Phosphorylated to AZT-triphosphate; mitochondrial uptake	Mitochondrial toxicity revealed *via* TCA cycle perturbations and lipid profile shifts	[187]

## 7 Metabolomics in antidiabetic drug discovery

Type 1 diabetes and type 2 diabetes are common metabolic disorders attributed to inadequate insulin secretion and insulin resistance, respectively [188]. Many families of antidiabetic agents have been discovered and marketed, including the biguanides, sulfonylureas, thiazolidinediones, DGAT-1 inhibitors, glucagon-like peptide-1 receptor agonists, dipeptidyl peptidase-4 (DPP-4) inhibitors, and sodium-glucose cotransporter 2 (SGLT2) inhibitors [189]. However, many patients are resistant to these antidiabetic agents or suffer serious side effects [190]. Therefore, several unmet needs exist for developing new antidiabetic agents for treating type 1 diabetes, type 2 diabetes, and related comorbidities [191].

**Fig. 5:**
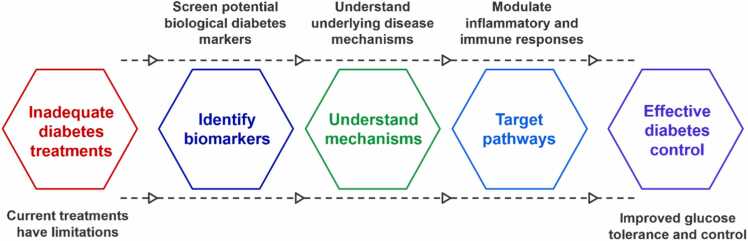
Metabolomics advances diabetes research and drug discovery.

Several natural products have been reported to have antidiabetic and anti-inflammatory activities [192]. However, the chemical diversity, bioactivity, and molecular mechanisms for antidiabetic agents isolated from traditional Chinese medicine were unclear due to the absence of systematic studies of activity-activity or ligand-target networks from unbiased studies [193]. Metabolomics contributed to the identification of central targets and essential reactive mechanisms for drug action in glucose and/or lipid metabolism, mitochondrial function, and energy metabolism [194]. This review also provides a timely and comprehensive overview of the recent discovery and application of the effects and molecular mechanisms of natural products on glucose and lipid metabolism for new antidiabetic drug candidates by using pharmacology, cellular studies, and metabolomics [195], [196], as schemed in (Fig. 5). In total, 14 natural products were reported to alleviate the symptoms of diabetes and inflammation to activate AMPK, PPARα, PPARγ, IRA-1, PI3K, IRS-1, GCR, eGP, and LXRα, which are the key targets related to ado-Met and/or glucose and lipid metabolism [197].

### 7.1 Metabolomics for diabetes research

Diabetes mellitus is a group of metabolic diseases characterized by hyperglycemia [198]. The importance of reducing the risk factors associated with diabetes mellitus and its complications has led to the development and continuous improvement of phenotyping tools to better understand the factors associated with the disease [199]. Different strategies have been used to study diabetes and its associated complications [200]. Among these strategies, metabolomics is one of the primary techniques for understanding in detail how multiple factors, including environmental, genetic, and microbiome interactions, contribute to the occurrence of the disease [201]. This powerful tool has allowed the screening of potential biological markers, which aid in diabetes diagnosis and the development of more effective drugs by studying the metabolites affected in diabetic rodents [202]. The present review shows the application of metabolomics in preclinical models treated with drugs with antidiabetic activity [199].

Metabolic syndrome and type 2 diabetes mellitus are both growing problems worldwide [203]. It is, therefore, vitally important to understand the underlying mechanisms and develop new therapeutic strategies [204]. Inflammation and metabolism are inextricably linked, and both have an impact on the development of this disease [205]. However, distinguishing between the various source organs of inflammation can be very challenging [206]. On the other hand, recent advances, particularly in metabolomic and metagenomic techniques, may provide us with new tools to understand the key pathways implicated in hyperglycemia [207].

The inhibitory phosphorylation of the insulin receptor tyrosine can also lead to the suppression of the translocation of GLUT-2 in the pancreas, GLUT-4 in muscle, and both GLUT-4 and GLUT-2 in the liver, resulting in an inability of blood glucose to enter cells [208]. Previously, work was performed using a compound that increased the levels of blood glucose and exhibited molecular inhibition of kinases and phosphatases of the insulin signaling [209]. This compound provides a new concept in antidiabetic therapy and is superior to the conventional pharmacological treatment in cases of failure of conventional therapy [210]. This molecule, available at different phases of clinical research, reduces side effects and improves safety [211]. The level of interest in measuring insulin and/or phosphorylated insulin receptors as a promising potential biomarker has increased considerably, resulting in a heightened requirement for a standardized and reliable method of measurement [206].

### 7.2 Targeting metabolic pathways in diabetes

Hyperglycemia, the defining feature of diabetes mellitus, is a common disorder characterized by elevated plasma glucose concentrations [212]. Effective metabolic control of type 2 diabetes is achieved through a combination treatment that decreases insulin resistance and enhances insulin secretion, thereby improving glucose tolerance [213]. Insulin-sensitizing agents increase peripheral glucose uptake and inhibit gluconeogenesis in the liver [214]. To target metabolic pathways in diabetes, drugs should not only target the insulin-signaling pathway in the liver and muscle but also modulate the inflammatory response, the function of the immune system, and enhance a microorganism-associated molecular pattern-triggered physical defense against inflammation [204].

Genomic-consensus-driven pharmacodynamics must provide single, high-potency drug candidates that inhibit or enhance bidirectional signaling pathways backed up by corresponding cellular and systemic signaling [215]. The currently efficient method for searching for these interactions involves multi-omics analysis [216]. Metabolomics, at the cutting edge of omics technologies, is technically the preferred method for linking drug or gene expression patterns with metabolic pathway alterations and signaling pathway reconstructions [2]. Starting from there, label-free metabolome assessment technology would enable simultaneous label-free pharmacokinetic structure-activity relationships, as well as toxicokinetic structure-activity relationships, for label-free efficacy optimizations [217]. Having quantitative fingerprints and alpha-bond fragmentations, this technique is best suited as a drug discovery method [218].

## 8 Metabolomics in anti-inflammatory drug discovery

Metabolic profiling may effectively distinguish mechanisms of anti-inflammatory activities [219]. Additionally, it provides a sensitive tool to detect the dose-response relationships underlying drug pharmacodynamics [220]. The application of metabolomics to pharmacodynamics is especially well-suited for the long-term, variable effects of chronic inflammatory diseases, such as inflammatory bowel disease (IBD) [221]. Metabolomic evidence requires only the delineation of the onset, magnitude, and duration of the drug response from clinical studies, which can be obtained by baseline or longitudinal studies of patients on a stable drug dose under the guidance of their physicians [222]. This evidence may help to ensure that patient drug responses are aligned with related metabolic events [1], [10]. The high time resolution of metabolic profiling diagnostics may also allow for the determination and management of mitigative drug regimens in collaborative drug repurposing approaches [223]. This approach may allow for the reduction of drug side effects by offering the lowest therapeutic dosages necessary [111]. The highly sensitive and specific nature of metabolic markers may provide immediate feedback on patient responses in adaptive drug dosing [17]. This can guide patient genotype-based dosing strategies [220]. However, phenotyping techniques currently face a significant bottleneck in technological growth, hindering the identification of drug metabolites [224]. Metabolomic elucidation of xenobiotic metabolic networks would overcome a confounding limitation in the bioavailability, bioactivity, and metabolism of known metabolites [225]. However, in the case of mass spectrometric data, this is an increasingly attainable goal. Residuals of such metabolic studies include acquiring the ideal patient baseline, postprandial, and longitudinal blood and fecal collection times, some of which may be an unconscionable invasion of privacy [226].

Inflammation is the body’s immediate response to infections and injuries, characterized by redness, swelling, heat, pain, and altered function [227]. Older cell types can also suffer changes known as cell senescence [228]. Chronic inflammation is linked to six of the twelve principal causes of mortality globally and is additionally resistant to anti-inflammatory treatment [229]. Metabolomics provides an independent and comprehensive, solutions-focused approach to the polymorbidity of aging and the pathological metabolic burden associated with healthy functioning in aging [230]. Chronic inflammation is responsible for disabling conditions, including arthritis, cardiovascular disease, cancer, and diabetes [231]. Moreover, the majority of patients who were hospitalized with other severe illnesses also show increased acute-phase proteins in their blood and tissues, which contribute to other chronic comorbidities [232], [233]. The core clinical chemistry measurements that are informative of the effectiveness of a treatment have now been identified as biomarkers of immune exacerbation [233]. Metabolic phenotyping facilitates a deeper understanding of the mechanisms by which different classes of immunosuppressants and anti-inflammatory drugs exert their effects [234]. The quantitative and functional information obtained from the detailed molecular characterization of the disease will enable better stratification of patients for the clinical application of effective new treatment regimens [29]. Metabolomics could also uncover new targets relevant for multiple immune disease processes [6].

### 8.1 Metabolomics for inflammatory biomarker discovery

Metabolomics coupled with toxicogenomics can help advance the discovery of urinary metabolite biomarkers of inflammation on a wide scale. It can link to the progression of sterile inflammatory diseases associated with the formation of crystals or to pathways of pathogen induction in infectious diseases [235]. They can also be used to further the understanding of the toxicology of crystalline antigens and pathogen-associated molecular patterns, to help establish the broad difference between human response and animal models, and to give insights into the potential root cause of side effects that organizations would wish to avoid, detect, and manage in their patients [236]. Furthermore, working toward the development of adverse outcome pathways for inflammation, these methods could offer new paradigms for the replacement of *in vivo* animal experiments and support the design of effective, low-variability, and inexpensive, high-throughput assays for testing the inflammatory potential of new compounds [237]. In this review, current knowledge of the roles of inflammatory patterns and the metabolic-associated morphologies observed in well-known defenders and attackers in living organisms is presented within an evolutionary context. To date, metabolomics has been minimally applied to defense and immune responses [238]. However, in many organisms, the system, triggered by the pattern recognition receptor, functions exquisitely and continually during host-pathogen confrontations to screen pathogen virulence factors [238]. Metabolic phenotyping of the acute inflammation model, along with associated serum and *ex vivo* human kidney tissue metabolomics, provides a basis for proposing potential strategies for metabolite biomarker discovery associated with continuous pattern screening by carbohydrate-binding defense molecules, which recognize carbohydrates bound to crystalline pathogen-associated molecular patterns [239].

### 8.2 Metabolomic strategies for anti-inflammatory drug development

Inflammation is one mechanism through which the mammalian body attempts to repair itself and maintain homeostasis [227]. Nonetheless, disruption in the regulation of the inflammatory response, characterized by prolonged, unresolved inflammation, is associated with various disease states, including cancer, obesity, type 2 diabetes, cardiovascular diseases, and neurodegenerative diseases such as Alzheimer’s and Parkinson’s [212], [240]. Many anti-inflammatory drugs are already on the market, but none address the majority of the underlying triggers of the response [241]. The discipline of metabolomics can provide a method for gaining more insight into the poorly understood inflammatory pathways, leading to the potential for defining anti-inflammatory clusters of metabolites that are influenced by different anti-inflammatory drugs [242]. Metabolomic studies for assessing and discovering anti-inflammatory drugs have been limited in number and often focus on targeted metabolite profiling, primarily due to challenges in identifying and quantifying endogenous metabolites [138]. With the development of global metabolomic profiling tools and databases, unbiased metabolomic studies are expected to be extremely valuable for anti-inflammatory drug research and development in the future [243]. Metabolomic studies may enable classification through pattern recognition and the prediction of uncharacterized compounds based on their chemical shifts using Nuclear Magnetic Resonance Spectroscopy or their fragmentation patterns using Mass Spectrometry [244], [245]. Metabolomic analysis using NMR and MS provides a wealth of quantitative information for potentially hundreds to thousands of metabolites, which can be utilized for systems biology, including the identification of mechanistic pathways [246].

## 9 Analytical approaches for computational metabolomics

### 9.1 Nuclear magnetic resonance (NMR) spectroscopy

**Fig. 6:**
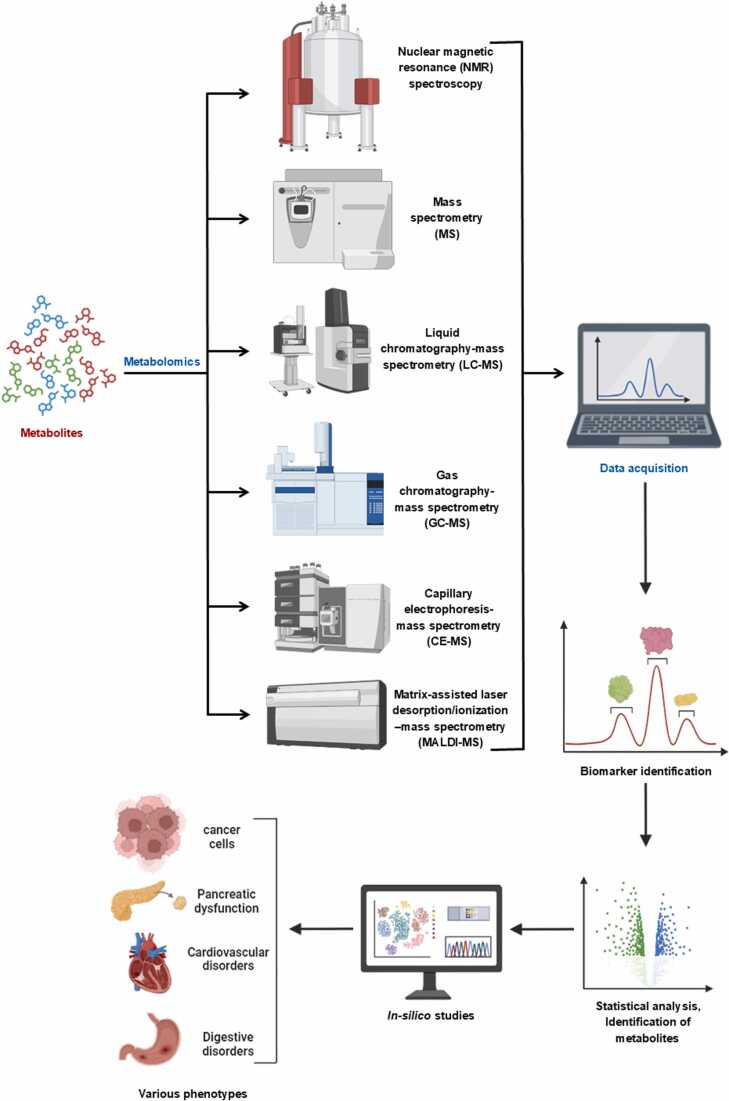
Variable metabolic phenotyping techniques. This figure was partially generated by Biorender (BioRender.com).

NMR is a powerful observational technique employed to evaluate individual biomolecule dynamics at the structure determination step, including binding interactions, post-translational modifications, conformational changes, and chemical kinetics with atomic-level detail [244]. ^1^H-NMR detects survival metabolic phenotypes and metabolite components during metabolic pathways under physiological status in organisms, such as cells, tissues, body fluids, and biofluids [247] (Fig. 6). If high-definition spectra with quick quantification are required, for example, serum metabonomics/metabolomics, ^1^H-NMR is perfect for broad-scope survey and metabolite assignment due to fast sample preparation and inapplicability for chromatography [248]. A modern NMR spectrometer facilitates management with multi-nuclear capability, thanks to the feasible integration of bioinformatics and NMR, along with computational and chemometric tools [249]. NMR provides mostly good line shapes of target signals, but not every metabolite [250]. Non-multiplex means that an issue arises due to sensitivity concerns resulting from the low inherent NMR sensitivity of the isotopomer number and atom/mark [251]. The high-field achievements with very high-field and ultra-high-field magnets are enhanced under low temperatures using cryoprobes for increased sensitivity [252]. A High-resolution metabolome has been extracted using HR NMR with advanced software for the metabolite profile [253]. A practical and unique non-invasive NMR metabonomic technique that employs a small living body was developed by applying a non-heating magnet [249].

### 9.2 Mass spectrometry (MS)

Mass spectrometry (MS) is the technique of choice in metabolomics for the identification and quantification of large numbers of metabolites that define the functional state of cells, tissues, organs, and organisms [111]. Here, we discuss MS-based methods used for the class separation, detection, and quantification of large numbers of metabolites in biological samples, and how they are applied to determine the metabolic basis of drug activity [254]. The four methods of mass spectrometry that we examine are (a) liquid chromatography/ultraviolet (LC/UV) and mass spectrometry (LC/MS) if coupled with electrospray ionization (ESI) [255] (Fig. 6); (b) gas chromatography (GC)/MS [256]; (c) shotgun lipidomic LC/MS if coupled with ESI [257]; and (d) matrix-assisted laser desorption/ionization (MALDI)-MS imaging [258]. Other works have shown that the comprehensive detection of metabolites essential in cellular processes, such as with LC/ESI-MS, is important, as depicted by its importance in current drug searches [259].

In LC/ESI-MS, a sample is separated by normal or reverse phase HPLC or ultraperformance liquid chromatography (UPLC) (accomplishing both phase separations in 10–100 μL) and directed into the ESI source [260]. In ESI, the droplets in the expanding column effluent are size-reduced, desolvated, and ionized with high efficiency by a flow of heated nitrogen [261]. The ions have high charge numbers due to their high surface charge: either the solvent evaporates, or the ions are desolvated through interactions with nitrogen gas [262]. The significant charges ensure that ions can be effectively steered by electromagnetic fields [263]. To operate ESI, ions are generated at an electrical potential of between 2 and 5 kV, with droplets coming from the separating chromatography system at a flow rate of 100 μL/min [264]. LC/UV can be installed by adding a standard UV diode-array detector, which co-elutes all metabolites in the chromatogram with high quality [265]. The integration of chromatograms across UV and the mass spectrometer demonstrates the superiority of extracting peak areas from chromatograms [266].

### 9.3 Gas chromatography-mass spectrometry (GC-MS)

Among the mass spectrometer techniques, GC-MS is the most mature [267]. It is an excellent choice for volatile and semi-volatile compounds due to its simplicity, high resolution, selectivity, and chromatographic repeatability [268]. In GC-MS, an interference-free mass spectrum results from a separation process, which is important in structure elucidation [256] (Fig. 6). On the other hand, a significant limitation is that the compound to be analyzed must be sufficiently volatile, allowing for simple chemical or biochemical derivatization to improve volatility [269]. Polar and thermally labile compounds may also chemically react during the derivatization process, causing inconsistent levels *in vivo* [270]. The alternative for the analysis of unmodified primary compounds and the detection of secondary metabolites by liquid chromatography-mass spectrometry has been increasingly adopted in the last decade, mainly due to the growing efficiency of HPLC [114].

The combination of gas chromatography with mass spectrometry, specifically multiple reaction monitoring, has become a primary approach for target metabolic profiling [271]. Advanced work has enabled the ability to perform metabolite profiling without making prior assumptions [44]. This capability was achieved through the measurement of patterns of gas separation, which is one aspect of chromatography; temperature programming is another aspect of chromatography; and the mass-to-charge ratio is one aspect of mass spectrometry [268]. Gas-phase ions are created with a signal related to the concentration of the metabolite and the mass-to-charge ratio of interest [256].

### 9.4 Liquid chromatography-mass spectrometry (LC-MS)

Liquid chromatography (LC) is coupled with mass spectrometry (MS) for the identification and quantification of low-molecular-mass metabolites [272]. This can include drugs and their metabolites in pharmacokinetic studies [273]. LC-MS can be used for profiling the metabolic fate of medicinal drugs and potentially toxic metabolites of carcinogens from biologically compliant samples involving radioactive drugs [18] (Fig. 6). The inherently high resolving power and sensitivity of LC-MS over the standard post-labeling methods allow the study of drug-associated proteins and DNA [274]. Specific ligands, antibodies, or nucleotide probes can be employed in combination with LC-MS to study drug metabolite adducts, which involve cellular macromolecules [275]. High metabolic throughput provided by LC-MS is particularly useful for preclinical drug testing with established cell lines [276]. This could be employed in high-content studies for gene expression, signal transduction, protein phosphorylation, and many other relatively new areas that use modern non-radioactive MS methodology [29]. Finally, LC-MS could be employed for the bioanalysis of samples obtained during the initial stages of clinical trials [277]. This encompasses exposure to clinically relevant doses and the identification of any potential toxic metabolites that may endanger the safety of newly developed drugs [278].

### 9.5 Capillary electrophoresis-mass spectrometry (CE-MS)

Capillary electrophoresis-mass spectrometry (CE-MS) is a powerful complementary method, as CE has its own unique attributes [279]. It is a non-biased separation method that can analyze basic analytes [280]. As a selective separation technique, CE can provide efficient and deep analysis for very complex mixtures of metabolites [281]. Especially, CE-ESI-MS is well-suited for detecting small metabolites that are not amenable to GC- and LC-MS [114] (Fig. 6). Therefore, metabolic profiling with CE/ESI-MS has proven particularly useful for highly selective, in-depth metabolomic studies [111]. This method allows for the quantitative and qualitative determination of metabolites from diverse chemical classes, including carboxylic acids, amino acids, mono- and disaccharides, phosphate esters, and amines [282].

CE-MS possesses unparalleled separation power, is highly sensitive, and provides high-throughput, rapid analysis of both charged and neutral solutes [282]. Capillary electrophoresis using electrospray ionization tandem mass spectrometry (CE-ESI-MS) has overcome several limitations of individual CE or ESI-MS [283]. CE-MS is a high-efficiency separation mass spectrometer. CE-MS has a wider solute dynamic range; CE can resolve many components that are difficult to analyze by LC-MS [284]. Using non-aqueous solvent systems with increased solubilities has further enhanced the scope of CE for mass spectrometric characterization of highly hydrophobic molecules [281]. In recent years, novel platforms combining electrospray and atmospheric pressure chemical ionization with CE have enhanced metabolomic analyses [111]. Capillary zone electrophoresis (CZE) is a widespread approach for analyzing acidic and basic metabolites, as well as hydrophilic solute protocols for neutral molecules [285].

### 9.6 Matrix-assisted laser desorption/ionization–mass spectrometry (MALDI-MS)

MALDI involves the ionization of matrix-embedded analytes by a laser for MS determination (Fig. 6). It is frequently used in imaging mass spectrometry to map metabolite distributions spatially [340]. MALDI-MS is an invaluable technique for mapping drug distribution and tissue-specific metabolism and metabolic traits [286]. It facilitates computational modeling of spatial metabolic dynamics and provides a framework for pharmacological profiling in preclinical models [287]. This approach is known for its high throughput, simple sample preparation, which is compatible with tissue imaging and spatial metabolomics, and its high performance for large biomolecules (lipids, peptides) [288]. However, it is limited by its matrix interferences, which affect low-mass ions, its restricted quantitative performance, and its lower resolution compared to that of LC-MS for small metabolites [289].

### 9.7 Shotgun lipidomics

Shotgun lipidomics is a direct infusion, high-throughput approach that enables mass spectrometric analysis of complex lipid extracts without the need for chromatography [290]. The principle of this method is its combination of electrospray ionization and tandem mass spectrometry for high-throughput and broad lipid analysis [291]. The computational framework is based on the resolution of overlapping isotopic peaks and *in-silico* spectral libraries, allowing for the accurate identification and quantification of lipids across multiple lipid classes [292]. Sophisticated algorithms simulate fragmentation and ion suppression to increase structural resolution [293]. Furthermore, it is possible to quantitatively relate perturbations in lipid molecular species to metabolic phenotypes using shotgun lipidomics in concert with multiscale systems biology strategies [1]. Its speed, depth, and convenience in various computational pipelines are pivotal for large-scale metabolomic studies in high dimensions [294].

## 10 Future perspectives

In the future, research studies with a greater number of biological replicates and improved industrial collaborations should apply equitable data standards to facilitate validation, normalization, and analysis processes [8], [295]. Adequate and rigorous validation work is necessary, particularly including linear models and fold change analyses for data highly skewed [296]. Combining metabolic profiling and expression-based profiling in the human disease environment is also thought likely to provide a more comprehensive overview of disease and phenotype (the metabolically labile phenotype is invoked) [222], [297]. Subsequently, a detailed understanding of the metabolic pathway and phenotype-involving mechanisms will be necessary to advance metabolomics techniques further. The powerful multifactorial level study designs, which favor the multifactorial analysis of a model system from different viewpoints, can provide essential information on aspects such as biological baseline metabolic variability, confounding biological effects, and variability due to different sample matrix natures, among others [298]. Anticipating the progress brought about for the next generation of NMR technologies, the development of more advanced ^1^H-NMR and MS-based technologies for improved sensitivity, resolution, reproducibility, and speed in obtaining results, in any laboratory and/or collaborative setting, will be critical for surmounting the existing barriers [299].

## 11 Summary and outlook

Herein, we introduce a metabolic phenotyping approach that addresses the investigation of metabolic alterations caused by exposure to drug compounds in appropriate model systems. We support that there is an opportunity for a broader inclusion of such technologies in drug discovery, having provided an overview of how metabolic phenotyping and metabolomics methodologies and related research have contributed to the discovery of new drug compounds in the last few years, making a special emphasis on those with activity in the areas of cancer, diabetes, inflammation, and metabolic and infectious diseases. In doing so, we discussed the mechanisms behind the observed metabolic alterations in the context of potential sites for therapeutic links between metabolites and lipid mediators, advancing research, including within cell disease or infection models, to identify new molecular targets for intervention and new molecular mechanisms of disease. Additionally, this non-targeted approach has the potential to contribute to a reduction in the existing rates of attrition in the later phases of the drug development process, as metabolic readouts are not easily evolvable by pathogenic organisms or model cell lines in response to the effects of drug inhibitory actions.

## 12 Expert opinion

Computational metabolomics, which combines multiscale *in silico* strategies with high-throughput analytical systems, has reached an impressive state of the art, poised to revolutionize drug discovery and biological research. The integration of metabolomic profiling with molecular docking, machine learning, and network modeling not only expedites the discovery of bioactive compounds but also provides an elaborate mechanistic understanding of the anticancer, antiviral, antidiabetic, antioxidant, anti-inflammatory, and antimicrobial properties. It has the potential to connect the omics data and functional therapeutics through a system-level understanding of metabolic pathways. With advances in data integration tools and computational power, these models will contribute to the progression of personalized and precision medicine. However, challenges in terms of data standardization, the interpretability of AI models, and clinical validation are significant barriers to addressing broader translational implications.

## Funding

This work was supported and funded by the Deanship of Scientific Research at Imam Mohammad Ibn Saud Islamic University (10.13039/501100008840IMSIU) (Grant number IMSIU-DDRSP2502).

## Declaration of Generative AI and AI-assisted technologies in the writing process

During the preparation of this work, the authors used Grammarly for editing and English proofreading. After using this tool, the authors reviewed and edited the content as needed and took full responsibility for the content of the publication.

## CRediT authorship contribution statement

**Mohamed S. Nafie:** Writing – review & editing, Writing – original draft, Visualization, Conceptualization. **Mohamed K. Diab:** Writing – review & editing, Writing – original draft, Visualization, Conceptualization. **Abdelghafar M. Abu-Elsaoud:** Writing – review & editing, Visualization, Supervision, Funding acquisition, Conceptualization.

## Declaration of Competing Interest

The authors declare that they have no known competing financial interests or personal relationships that could have appeared to influence the work reported in this manuscript.

## Data Availability

All data generated or analyzed during this study are included in this published article.
